# Cases of the Principal Diseases of the Female Organs of Generation

**Published:** 1809-01

**Authors:** John Roberton

**Affiliations:** Surgeon, Edinburgh, Author of the Practical Treatise on Gleet, Leucorrhœa, and Obstinate Sores


*Cases of the principal Diseases of the Female Organs of Generation.* By John Roberton, Surgeon, Edinburgh, Author of the Practical Treatise on Gleet, Leucorrhœa, and Obstinate Sores.

In considering diseases of this kind, as the most important of them are connected with the catamenia, it may be proper first to turn our attention to that subject.

Menstruation, as Dr. Cullen seems to suppose, does not depend on any particular force or action of the uterine vessels, vessels themselves, but it happens in consequence of a particular change on the whole system at a certain time of life, different in different countries, and in different individuals of the same country; and the uterine arteries seem naturally constructed so as to assume the power of secreting the menstrual fluid at a certain time, and only under certain circumstances, although in the interim, the general health of the individual has undergone no apparent change. When, therefore, suppression or retention of the menses occurs, the treatment ought entirely to be indicated by those symptoms, or that general state of the body, which seemed to constitute the principal, perhaps the only cause of these complaints. I mean, that as menstruation depends on a general state of the system, all its derangements are to be considered as resulting from derangements of the system in general, and that therefore, in the medical treatment applicable to them, general applications are to be adopted, and topical ones, at present almost entirely resorted to, to be avoided. General remedies are indeed sometimes resorted to, but they are, in my opinion, altogether inapplicable. If it arises from debility, the cold bath with general stimulants are to be had recourse to; if from plethora, friction, depleting medicines, blistering, and abstraction of blood in such quantities as may be best suited to the strength of the patient, must be preferred.

Indeed, Cullen's Theory of Menstruation seems equally erroneous with his opinions respecting the nature of several of the diseases of the generative organs. Allow him his doctrine of spasm, and he will explain any thing; but deprive him of that method of extricating himself, which he indiscriminately has recourse to when in difficulty, and his hypotheses of these, as well as of many other diseases, must fall. It seems to me, that we may, with equal justice, and with as great probability, ascribe every operation in the animal body to the interference of spasm, either in a state of health or disease. Even were we, however, to adopt that plan, our reasoning in support of such hypotheses could not be less vague, than that which Cullen often has reconrse to in support of his favorite doctrine. In short, his Theory of Menstruation seems to me equally absurd with that of Aristotle, who supposed it to depend on the influence of the Moon, or that of the chemical physicians, who believed it to be caused by fermentation.

Habitual menorrhagia, chlorosis, (whether from suppression sion or retention of the menses) leucorrhœa and dysmenorrhœa, although in appearance different from each other, seem nearly, or indeed wholly, to arise from the same cause. All the causes of menorrhagia, for instance, assigned by Cullen in his First Lines, such as a continuance of full and nourishing diet, much strong liquor, intoxication, violent shocks of the whole body from falls or contusions on the lower belly, violent exercise, violent passions of the mind, excess in venery, particularly during menstruation, a costive habit, &c.; frequent abortions, frequent child-bearing without nursing, difficult tedious labours, living much in warm chambers and drinking much warm enervating liquorss, such as tea, &c. are evidently calculated, in opposition to his own opinion, to induce, directly or indirectly, a state of local or general debility previous to the appearance of the disease; consequently I cannot with him consider it as an active hæmorrhage.

Cullen's mode of considering amenorrhœa or chlorosis seems very judicious; but, in his practice, there is a great falling off; for the remedies he recommends in suppression of the menses, are either totally incapable of permanently removing general or local debility, or calculated in a great measure to increase the very debility which he wishes to remove, such as purging, mercury, &c.

He forbids tonics and cold bathing in suppression of the menses, as they appeared to him of ambiguous effect.

In advanced stages of all these complaints, the most depressing debility is experienced on the very slightest exercise being used, and even this takes place without motion, while the pulse is uniformly feeble and irregular.

While enumerating these diseases, I may take notice of another complaint, which, in a practical point of view, resembles those I have just mentioned. I allude to that distressing irregularity in menstruation which very commonly torments the patient for years, but more frequently only a few months previous to the complete disappearance of the catamenia.

Whatever may have been the original cause of what is called dysmenorrhœa, that is, when the catamenia flow with difficnlty and are accompanied with great pain, we know debility to be in general present to a great degree when that disease is violent.

Cullen thinks, as usual, that it depends on spasm of the extreme vessels of the uterus; perhaps this may exist along with it, but I do not think that this is the cause of it; nor do I think his practice of prescribing opiates, though it remove move such spasm, can remove the disease. Dr. Hamilton, Professor of Midwifery, also has the utmost faith in the operation of hyociamus, which acts nearly on the same principle.

Now we know, that both these substances are very useful in removing the pain occasioned by the disease, but from our numerous disappointments in the cure of these affections by such means, we must long have been convinced of their inutility, farther than as they procure mere temporary ease. The same diseased state of the parts still continue, and the only service derived from these substances is, perhaps, relief for the time; but when the next menstrual period arrives, all the former symptoms, perhaps in an aggravated degree, recur, and the harrassed and enfeebled patient at length sinks under her accumulated sufferings by the supervention of dropsy in one or other form, probably by consumption of the lungs. Practising with the remedies recommended by Cullen, or indeed with any remedies, (if they be suited to his general mode of reasoning in these complaints) will, I venture to assert, be in every case unsuccessful, except in very recent cases where the general health and strength have been unimpaired. When the disease has in a great degree deranged the general health, Cullen seems to think it incurable. The only remedies he then recommends are, external and internal astringents, cold bathing, and chalybeates, of which, with similar applications, I have repeatedly given, and that to the greatest extent, without, in such cases, deriving any permanent advantage from them.

The cases which I have lately seen of what female patients call lumbago, are too numerous to be particularised; many of them entirely depending on a diseased action of the generative organs, and capable only of being relieved by the removal of such disease. Although I was aware that pains, in many respects similar in their nature to those of lumbago, are very common in far advanced and very bad cases of leucorrhœa; yet, till lately, I never have met with cases of leucorrhœa, where the discharge had always been of small qnantity; indeed, in some cases, scarcely perceptible; and in these, the most acute pains of the loins accompanied it. Such a state of disease has not only been considered as lumbago, chronic rheumatism, gout, &c. but treated as such, and the unfortunate patient has been obliged to undergo, in vain, every variety of treatment recommended by authors for the removal of such complaints; when, as might have been expected, ed, want of success attended their labour, even the horrible supposition of lumbar abscess being the cause of the pain, has been entertained, and bleeding, blistering, with the use of setons, have been obstinately persisted in for its removal. What might not seem very extraordinary, however, the patient remained uncured; but when such means were applied as were suitable for the removal of the complaint, the rheumatism or lumbar abscess, or whatever term they chose to give it, entirely disappeared.

Thus, by considering these complaints as totally different, and recommending entirely a different mode of treatment for each, the practitioner not only fails in that success which is wished for, but renders their natural simplicity extremely perplexing.

If one or more of these complaints be brought on by long continued, though slight, leucorrhœa, our attention being principally paid to this last, and a complete removal of it effected, almost all the former affections are completely removed.

The treatment of these complaints has been various, regulated rather by accident or the whim of the moment, than by the success attending it, or by any fair mode of reasoning, when such has at all been attempted by the physician. In consequence of this complete want of success, these complaints have long been tacitly considered as incurable, unless some favourable change accidentally took place in the constitution of the patient, which was all that either the physician or patient looked to for relief. Physicians, therefore, considering these diseases as incurable, instead of devoting their time to discover some successful mode of treatment, employ themselves in amusing their patients by assuring them that the various natural changes which, at certain periods of their lives, must take place in the system, will *probably* effect a removal of their complaints. Thus, in anxious expectation of such changes taking place, the patient's vigor of constitution is gradually yet surely wasted; and too often, without the arrival of the long and anxiously wished for relief, other diseases, consequences of the first, attack them, which for the most part, only terminate with their miserable existence.

It is by the reasoning adopted by authors respecting the nature, &c. of complaints that the treatment in them must flow; and in proportion as this is right or otherwise, our cures, except by some accidental occurence, must be few or numerous.

Although during the early stages of menorrhagia, if it occur, occur, which it often does, in stout plethoric persons, when the pulse is unabated in strength, when no apparent debility has been induced; in short, when all the other functions of the body seem unimpaired, and even when blood-letting is indicated by the apparent fullness and inflammatory action of the system, the greatest caution, even at this period, ought to be observed in adopting general blood-letting for its removal. Even the exhibition of medicines that may ultimately induce debility, ought to be resorted to with nearly equal caution. For even although these means may, in almost every case, remove the morbid discharge, I have often observed, that, after such treatment, it was long before the patient recovered her usual strength, and she remained often for years subject to returns of the menorrhagia from the very slightest causes. But when such practice has been adopted in weakly and debilitated habits, (for it is too often indiscriminately applied) the system is not only left in a dreadfully debilitated state, liable to almost continual flooding, but the most obstinate and troublesome cases of leucorrhœa that I have ever met with, have been brought on after the application of such means. The remedies then which Cullen recommends are either hurtful, inactive, or of a trifling nature, and upon the whole, by no means suited to the removal of such complaints. He forbids the use of all medicines that may irritate the parts. I think, however, that what I have to state will completely prove that such remedies only as Cullen thinks would irritate the uterus, are calculated permanently to remove such diseases; and that the chalybeates, &c. if deemed necessary along with such medicines, may be useful, but never can, except in the very slightest cases, effect a cure.

If during these complaints, which does not often happen, the pulse indicates inflammatory action, and the patient happens to be of a full plethoric habit, should cantharides be prescribed, I grant that it would require no difficult calculation to foretel what would be the result. Benefit must be evidently sought for from very different treatment.

In all these complaints, however, I believe the only medicines that can be employed with decided advantage, are those of a stimulating nature. Food and drink, as well as medicines, ought all to be considered in this way. In these affections, the uterine vessels are in a great state of disease; but it appears to me that the general habit of body has been and is equally deranged. The medicines therefore fore to be employed are such as will sufficiently affect the whole system, and the generative organs as a part of the whole.

It is of importance to observe, that a great proportion of women have been taught to believe that leucórrhœa is a natural discharge, the existence of which is, for the most part, absolutely necessary to the preservation of their health; and even if their health be already considerably impaired by it, the old and experienced matrons console themselves and others in the supposition, that to this discharge alone they owe the little health they possess. It is not therefore to be wondered at that women often so strenuously deny being affected by it.

### CASE I.

September 1807. An unmarried lady, aged 21, of a delicate habit of body, menstruated at the usual period, without any symptoms but such as are common on these occasions; and her health in every respect continued unimpaired fully two years; she was at that time attending a boarding school, and had exposed herself to cold and too much fatigue, in consequence of which her health suffered considerably. A few months after, her menstruation was greatly diminished in quantity, and although it always preserved the regular period, was attended with most excruciating pain, particularly on the first day after its commencement. Warm applications and sometimes friction, with an occasional laxative pill, were the only remedies she ever employed. From these, she derived only momentary relief; for, at each return of her menstruation, the same sort of applications were uniformly and necessarily had recourse to with similar partial benefit. In addition to her other complaints, she had for more than a year past, been affected with most distressing sickness, a day or two previous to the flow of her menses. This, however, in general, disappeared on their approach. About this time too her other complaints likewise subsided considerably; but several days elapsed before she recovered from a great degree of debility, probably induced by them. She never had any leucorrhœa.

When I was desired to visit this lady I found her very much emaciated, of a costive habit, with a fluttering and irregular pulse. Having been informed by her mother respecting the state of her complaints, I judged it improper, being unnecessary, to question her about them, as her feelings were of a very delicate nature. I explained to her

 mother mother what I conceived to be the nature of her complaints, and proposed to try the effects of the tincture of cantharides in removing them. In order to obviate the costiveness, I prescribed a pill composed of equal parts of the extract of hyociamus and aloes, and next day gave her tinct. cantharid. ʒß. aq. font. ℥vi.; a table spoonful to be taken thrice a day. I ordered her feet to be bathed in tepid water every night, and a flannel or thick cotton shift to be worn in preference to any other. This practice was continued, though sometimes rather irregularly, till next return of the menstruation; but there was no abatement in the violence of the symptoms above described. I now, with some difficulty, prevailed on this lady to continue the above prescription for another month. I desired her to take a good deal of exercise, on foot, and in carriage. During the use of it she never was sensible of any pain from the cantharides; and at her next menstrual period she did not, as formerly, experience sickness or retching. The other symptoms, however, remained as before, and the cantharides were continued.

Three successive menstrual periods recurred, and she suffered no uneasiness whatever. The only medicine which she then used was the pills of hyociamus and aloes.

In October 1808, she remained in good health, was much improved in her appearance, and her menstrual periods were regular and gave her no pain.

### CASE II.

A lady, aged 29, of a very delicate form, has been married twelve years and has had two children. Six years ago, a few weeks after the birth of the youngest, she was affected for the first time with leucorrhœa, which being uncommonly profuse, troubled her very much. If her mind was in any way agitated, which very frequently happened, her complaint was always aggravated in a great degree for several days. For the last three years she had suffered much from weakness in her loins, and from severe pains about the situation of the uterus, and the discharge had been of a thick yellow consistence. Bearing down pains too, as they are called, were often indescribably irksome to her, and from time to time, darting pains in these parts troubled her. She had likewise been, for about three years, affected with an eruption on different parts of her face, which was the complaint I was first consulted about, when, two months ago, I was desired to visit her. She did did not inform me of her being affected with leucorrhœa, I therefore prescribed a solution of the muriat of mercury in alcohol, to wash the affected parts with twice a day, which removed the eruption; it, however, soon recurred, and the same application was again had recourse to with the same beneficial effects.

On the 1st of December, 1807, I was informed of her being affected with leucorrhœa, and of the other symptoms connected with it, above described. I prescribed for her tinc. cantharid. ℥ß. aq. font. ℥vi.: a table-spoonful to be taken four times a day. She continued the use of this medicine, according to the above direction, fully a fortnight before she was sensible of any effect being produced on her urinary organs. She was likewise desired to employ cold lavation. A small augmentation of the dose of the cantharides, occasioned a good deal of pain during the night, while in bed, but this abated before morning.

On the 18th, she had continued the use of this medicine in sufficient doses to produce some degree of pain in voiding urine, and the pain was often considerable, particularly towards evening, which she partly ascribed to her want of caution in the use of the medicine. She thinks the discharge is less in quantity than formerly, and she is much less distressed with the bearing down and shooting pains across the loins. I have therefore desired the cantharides to be continued.

On the 24th, from incautious exposure to cold, she had for two days suffered a good deal from pains in different parts of her body, somewhat similar to a slight rheumatic affection, and she thinks the discharge is rather augmented in quantity. I ordered the cantharides still to be continued, but desired her to omit the cold lavation for a few days.

On the 26th, acute pain in the situation of the kidneys had troubled her since yesterday; the pain also in voiding urine had been troublesome, but the discharge was undiminished in quantity. I therefore ordered the cantharides to be continued.

On the 2d of January, 1808, the eruption on her face, formerly described, had considerably abated, but the leucorrhœal discharge was undiminished. Pains in her back were very troublesome. I have ordered the cantharides to be continued, and as the pains formerly mentioned had left her, I desired her to recommence the cold lavation.

On the 4th, she had suffered a considerable deal of un-

 easiness easiness from the cantharides during the two last days, particularly towards evening. She had likewise constantly felt a prickly sensation in her fingers, and a soreness in her face, and the pain also in the situation of the kidneys had been very troublesome to her. I ordered the cantharides to be continued only in small doses.

On the 10th, the discharge had greatly diminished since the last report, and instead of that peevishness, which once was so irksome to herself and distressing to others, she feels contented and happy, comparatively speaking, to what she had been for many years. I therefore ordered the cantharides to be continued.

On the 17th, the discharge was small in quantity: it, however, constituted the only symptom of her complaint; all her other distressing feelings having undergone a complete revolution for the better. The eruption on her face was now gone, and her appearance bespeaks a greater degree of health than she confesses she ever expected to enjoy. I desired the cantharides to be continued.

On the 20th, she suffered violent agitation of mind, in consequence of which she was very sick on the following morning. I however, desired the cantharides to be continued.

On the 22d, The discharge returned with very great violence, and the same kind of pain, which she originally felt in her back, likewise returned. I desired the cantharides to be continued.

On the 1st of February, a succession of unpleasant circumstances had agitated her mind since last report; the discharge was now worse than ever, and all her original complaints had returned in a violent degree. I ordered the cantharides therefore to be continued, and cold lavation to be used.

On the 3d, her complaints were again beginning to abate; yet her state of mind was by no means favourable for their removal. I desired the cantharides, &c. still to be continued, and three or four glasses of wine to be taken daily.

On the 8th, all her complaints had again abated except the discharge, and the eruption on her face was also completely gone. The cantharides however were continued.

On the 16th, from unavoidable circumstances, her mind was still very much distressed, and she invariably observed, immediately after such an occurrence, that she had a regular recurrence of the discharge. The cantharides were continued.

On

On the 26th, no alteration had appeared in the discharge since last report; but the pain in her back had greatly abated. The cantharides were still continued.

On the 1st of March, the discharge continued as above described. Three days preceding this she had struck her ankle against a table and bruised it considerably, and in one part abraded the skin. The inflammation of the wounded part had become violent, and I ordered it to be poulticed.

On the 4th, inflammation and pain of the leg had still continued to increase, I therefore desired her to omit the cantharides for a few days.

On the 10th, the sore was nearly well, but since she left off taking the cantharides the leucorrhœa had been worse. The cantharides however were still discontinued, and the sore dressed with simple ointment.

As, after this date, no material alteration in the daily reports of this case occurred, I deem it unnecessary to insert them. I may only mention, that this patient continued the use of the cantharides till the 1st of June, when the discharge had completely abated for more than two weeks. She was now, in every respect in a better state of health than she had been for several years, and the eruption on her face had disappeared. I desired her to continue the cantharides in small doses for a few days, and to use the cold sea bathing.

### CASE III.

A married lady, aged 32, eleven years ago, bore a female child, and soon afterward was affected with very profuse leucorrhœa. She had had no children since. She suckled the child for ten months, during which time she never recovered her former strength. The first appearance of her menstruation after having nursed the child, was of much smaller quantity than formerly, yet still it observed the regular periods. She had been informed by some sage matrons, that the discharge of leucorrhœa was natural to her, and that every woman had it less or more, and indeed adduced arguments in proof of its being necessary to the preservation of her health, and that she ran the greatest risk of her life if she attempted to stop it! Thus influenced, she never attached to its existence any consideration of importance, but attributed the pains in her back, which she began to be troubled with, particularly about three years after the commencement of the disease, to other causes than this. She was now affected with general lassitude, accompanied by hysteria, and was advised by her former medical attendant to go to sea-bathing quarters, which improved her general health very considerably, but she experienced no good effect from it in the removal of the leucorrhœa. Finding this the only remedy from which she derived any benefit, she had recourse to it regularly every season, but still the leucorrhœa continued in greater quantity, and she at length became gradually more debilitated, notwithstanding the sea-bathing. About five years ago, her menstruation recurred every three weeks, and in very small quantity at a time, and she was continually troubled with a distressing weakness in her loins. From time to time too she felt pains darting down her thighs, and soon after this an eruption of purplish coloured pimples, unattended by any pain, broke out upon the under part of her face, which resisted the effects of a variety of substances, given her for their removal. She was now advised by the surgeon who attended, to use bark and wine, which she did in great quantities, and he, at the same time, prescribed an injection, for the removal of the leucorrhœa; but although at first the use of the bark gave her a better appetite, neither they nor the injection had any effect in removing the leucorrhœa. She soon became disgusted with the bark and wine, yet she persisted in its use, being repeatedly assured that it would remove her complaint. Nausea and vomiting, however, at length followed every dose of it, and she laid it entirely aside. The leucorrhœa still became more troublesome, so that she was constantly obliged to wear cloths, and she at length endeavoured to reconcile herself to her hard fate. She was sometime ago advised to put an issue in her arm, for the removal of the eruption on her face, which had assumed a very unpleasant appearance; and, eager to embrace any chance of alleviating her complaints, she placed great dependence on this application. When I was consulted about this patient, she gave me the above history of her disease, with the means which had been unsuccessfully prescribed for its removal. I conceived, from having met with similar instances, that the smallness of the quantity, and the frequent recurrence, of her menstruation, as well as the eruption on her face, depended, perhaps entirely, on the leucorrhœa, or rather on that state of body which occasioned it, and that the issue in her arm could have no good effect. I therefore ordered her to heal it, and prescribed for her on the 16th of November tinct. canth.

ʒß. aq.

℥ß. aq. font, ℥vi. a table spoonful to be taken four times a day. She took one spoonful at mid-day, and before the evening, when she intended to take the second dose, she was affected with great pain in passing water; evidently in consequence of having taken a single dose of the medicine. She however took the second, and suffered considerable pain during the night. This, however, abated before morning, and she had taken the doses regularly during this day, the 17th, without any return of the pain till the 21st. Although she had regularly taken ʒij. each day of the tincture, she had not, except almost immediately after the first dose, experienced any pain, when she felt slight uneasiness with a more frequent inclination than usual to void urine. She thought the quantity of the discharge had abated considerably, and expected her menstruation to appear four days before, but it did not. She however experienced no inconvenience in consequence of this, and I desired the cantharides to be continued.

On the 23d, slight cough, with pain in the head, had troubled her for two days, and I desired her to take a small tea cupful of an infusion of chamomile flowers thrice a day. For the alleviation of the cough I likewise desired her to inhale the steams of vinegar and warm water morning and evening, and to continue the cantharides.

On the 25th, the above symptoms had disappeared, and her menstruation came on this evening immediately after she had been taking an airing a few miles in a carriage. The cantharides, &c. were continued.

On the 28th, menstruation had ceased, and she found the discharge somewhat abated. The cantharides were still continued.

On the 1st of January, 1808, she had, since last report, experienced a good deal of pain from the use of the cantharides, but this had never been so severe as on the first day, after taking only one dose of that medicine. She frequently experienced pains in her head with general debility, which proved very troublesome for a few hours. She had, in such states, obtained partial relief from the use of purgatives, and once or twice, when these failed, she derived great benefit from the use of a strong tincture of gentian root, these symptoms evidently having their origin in the stomach. The eruption on her face was now much less than formerly, which gave her great hope of recovery, as always, on former occasions, it became greatly worse, for nearly a fortnight after her menstrual period. She now took ʒiij. of the cantharides each day; which which produced very little uneasiness, and I have desired that medicine to be continued.

On the 7th, the discharge was becoming evidently less in quantity; but she complained of great weakness, and very little exertion fatigued her. She could now take the cantharides only in small doses, owing to its effects on the urinary organs, and her urine was very high coloured, when she was affected in that way by the cantharides.

On the 9th, she took rather too large a dose of the cantharides, which kept her very uneasy for several hours.

On the 11th, a great deal of membranous substances had come off since the preceding day, resembling a quantity of chaff floating in her urine. I desired the cantharides to be continued in smaller doses.

On the 19th, the discharge had abated gradually since last report, and she now felt greater inclination to walk abroad, than she had done since a short time after the commencement of her complaint. The cantharides were therefore to be continued in rather larger doses.

On the 20th, she was suddenly alarmed by an unexpected occurrence, and her complaint, almost instantly, became much worse. She now began to despair of getting well, and it was with some difficulty that I could prevail on her to continue the cantharides. She, however, at length consented.

On the 26th, since last report the discharge had gradually abated, and she was nearly in the same condition as before the 20th. Menstruation commenced this day. I desired the cantharides to be continued.

On the 2d of February, the discharge had almost entirely disappeared. She had taken too large doses of the canth. three or four times this day, and toward evening she felt severe pain in the situation of the uterus; but very little pain in passing urine. I however desired her to discontinue the cantharides.

On the 6th, the pain had abated and the discharge was scarcely perceptible. I however desired her to take the cantharides, but in very small doses, and likewise three or four glasses of wine in the course of the day.

On the 8th, the discharge had entirely disappeared, and the eruption on her face was much better, but it had not entirely disappeared. The cantharides were still continued in small doses.

On the 16th, the discharge had recurred in considerable quantity, attended by pain in the lumbar region. She could could not account for this, but, by another member of the family, I was informed that her mind had been considerably agitated for two or three days before. This agitation of mind has uniformly had the effect of bringing back the discharge in almost every case of leucorrhœa that I have met with, particularly in ladies who are easily affected in this way. The cantharides were continued.

On the 18th, the discharge was scarcely perceptible. The cantharides however were continued.

On the 24th, menstruation commenced, and she thought that the eruption on her face was worse than it had been for several months. As the discharge had not entirly abated, I desired the cantharides to be continued.

On the 10th of March, the discharge lately varied in quantity, but it had never been near so bad as it was originally. I desired the cantharides to be continued.

In the beginning of April, the discharge had gradually abated, and was now entirely gone. Cold sea-bathing, small doses of cantharides, nourishing diet and gentle exercise, were desired to be used for a few weeks.

On the 6th of June, there had been no return of her complaints, and the eruption on her face had disappeared along with the leucorrhœa except one single pimple, which I have no doubt will be entirely removed before the sea-bathing season has elapesd.

September, 1808. This lady continued free from all her complaints.

### CASE IV.

Mrs. ———, aged 35, a little delicate woman, and mother of two children, being poor, was at each birth obliged to endure many inconveniencies while in child-bed, and when last in that situation, nine years ago, she was almost immediately after delivery, actually obliged to employ herself at some kind of work for the support of her family. While making a sudden exertion she felt something about her loins give a sort of a crack, but she was not sensible of any great inconvenience from it, except that the lochial discharge, which was before moderate, now increased in an alarming degree. This terminated in almost constant and profuse flooding, which for several months after continued to distress her. During its continuance she was bled and purged most unmercifully, but without deriving any benefit from them; on the contrary, she was sensible of the daily diminution of her strength.

About

About two years afterward the flooding became less violent, though still very troublesome, and about three years ago, she observed in the intervals between its appearance, that there was a constant glairy discharge, in considerable quantity, and this, with the flooding, attended by excessive pain, which, from time to time still continued, and had reduced her very much.

For eighteen months past, the leucorrhœa had been excessive, pains in her back had likewise been very troublesome; cough and pains in the breast, with great general debility added to these, had indeed rendered her existence a burthen to her. There is too a parched dryness all over her skin, of which she complains very much.

On the 22d of December, 1807, I prescribed tincture of cantharides ℥ij. thirty drops to be taken in a little water thrice a day.

On the 25th, she had experienced considerable pain in passing water, on which account she had declined taking her evening dose.

On the 26th, the pain had ceased, and she thought the weakness about her loins less severe. No other change had taken place; I therefore ordered the cantharides to be continued.

On the 29th, the discharge had abated in quantity, and she felt no pain or uneasiness in her back. The flooding, which still continued, was not attended with so much pain as formerly. I still desired her to continue the cantharides in sufficient doses, to keep the parts in a constant but slight degree of pain.

On the 12th of February, 1808, she had continued to follow the above direction, and the leucorrhœa and flooding had completely disappeared. She had no pain in her loins; the parched state of her skin, with the cough, had left her, and she was in every respect perfectly recovered.

On the 20th of June following, she continued free from pain, and was in much better health than she had been at any time since the commencement of the above described complaints.

### CASE V.

Mrs. ——— aged 30, of a delicate habit of body, was married several months before her menstruation, and about two months after it commenced she became pregnant. During the fourth month of gestation she had an abortion, followed by a considerable flooding, which, though severe while it lasted, abated in a few months without without the use of medicines. Five years afterward she again became pregnant, and bore a healthy stout child at the full time She observed, for several months, that two or three days before and after menstruation, she had a thin glary discharge, but in the interval this did not trouble her in the slightest degree.

About the beginning of the year 1807, she again became pregnant; in the fourth month, during the frost, she fell down stairs, which was immediately followed by violent flooding, and in a few days after an abortion. The midwife who attended her, used much violence in bringing off the placenta, to which my patient attributed the excessive flooding that for nearly three months afterwards distressed her. Abstinence from every stimulating substance was, by her medical attendant, strictly enjoined, because they would irritate the parts, and increase her complaints; but affusion of cold water was plentifully applied. From this treatment she derived no benefit, and at length desisted from this application. The flooding spontaneously left her a month ago, and for a few days about that time she had a very great leucorrhœal discharge. Her complaint was now thought to be consumption of the lungs, and treated as such till the 26th of December, when I saw her for the first time.

For nearly four months past she had lost, almost completely, power over her right arm; pains in every part of her body, but particularly in her back, had been most excruciating, and for several years she had been affected with hysteria to an indescribable extent. Her bowels were very costive.

As she did not appear to me to have consumption, I conceived, from the symptoms of her complaint, that this state of extreme debility was intimately connected with the diseased action of the generative organs, and I immediately altered the mode of treatment. I ordered her half a pint of wine a day, and desired her to take such soups as the weak state of her stomach would admit. I likewise prescribed tinct. cantharides ʒß. aq. font. ℥vj. a tablespoonful to be taken four times a day, and an aloe pill to be taken occasionally.

On the 3d of January, 1808, menstruation commenced, attended as usual with considerable pain; the menstrual discharge was very black, and in large coagulated pieces. She thought, however, that her general health was much improved; and as she had suffered no pain from the cantharides, I ordered that medicine to be continued.

On

On the 6th, the globus hystericus, which was formerly so troublesome, left her, but instead of it she was affected with acute pain in her breast. Menstruation had left her, and slight leucorrhœa had succeeded it. The pulse was very feeble. I prescribed for the removal of the hysteric affection, fetid pills; two to be taken when the pain in her breast became troublesome, and the cantharides, &c. to be continued.

Till the 10th, the pills uniformly removed the pains of her breast, and her health was improving daily. Cantharides continued.

On the 17th, she became anxious to get well, and last night took a larger than usual dose of the cantharides, which, for several hours, pained her very much; I therefore ordered the cantharides to be omitted till that symptom abated.

On the 20th, she informed me, that about an hour after she had been so violently affected by the cantharides on the 17th, she perceived a plentiful discharge of very limpid matter from the vagina, which had continued ever since. I desired her to resume the cantharides.

On the 27th, the limpid discharge had gradually abated, and she was now able to take a good deal of exercise in her room. Cantharides continued.

On the 29th, the pain was again produced by the cantharides, and the limpid discharge began to increase in quantity. The cantharides were continued, but in smaller doses.

February 7. Menstruation commenced, but without any troublesome symptom.

9th. Menstruation had ceased, and she perceived that, at this period, it had been more natural in its colour, &c. than for many months. She now became disgusted with the cantharides, and refused to take them any longer.

26th. The leucorrhœa had gradually become worse for the last fortnight, and was now in greater quantity than it ever had been. Perceiving the impropriety of leaving off the cantharides, she agreed to recommence them.

29th. The pain from the cantharides was again produced, but there was no alteration for the better in her complaint. The cantharides were continued in smaller doses, with wine, porter, and the daily use of cold lavation.

March 5. Before her menstruation began, which happened this morning perfectly regular, and unattended by pain, the leucorrhœal discharge had nearly disappeared. The cantharides, however, were still continued.

12th. The

12th. The discharge was scarcely perceptible, and, in every respect, she had nearly recovered her strength. The cantharides were continued.

24th. The discharge was entirely gone; the cantharides, however, were continued in small doses for a week, and the use of cold sea bathing with nourishing diet, &c. during the remainder of the summer.

In October, 1808, she continued entirely free from all her complaints.

### CASE VI.

Miss ———, aged 22, stoutly made but very pale, and of a sickly appearance, when an infant, was affected with great pain in the under part of her abdomen. This, from her indistinct account of it, was mistaken for a *bowel complaint*, and treated as such; but she scarcely derived even momentary relief from any applications that were made. A few years after, she was better able to describe the nature of her feelings, and it was not till then observed that she was affected with leucorrhœa in a slight degree, with frequent darting pains all over the generative organs, which last sensation was equally sudden in its attack and in its departure. Her urine was of the colour and apparent consistence of thick porter, and she uniformly experienced partial relief after discharging a quantity of it. Sometimes for a day or two she remained free from pain; at other times several weeks, without much suffering.

Her menstruation commenced when she was eleven years of age, but this was only partial, for a year elapsed before it continued regularly. Since that time, her complaints have been more severe, and she has had more frequent returns of them than before the menstrual period commenced. Her menstruation has been very regular, but in astonishingly small quantity, and attended with very great pain about her back, and the lower part of her belly. While passing urine, it frequently stopped suddenly, and then she felt considerable pain, with a sort of convulsive shuddering all over her body. When this left her she felt, for several hours, a general lassitude, and sometimes considerable sickness, and within these few months, she was seldom free from the most acute pain. She had used a great variety of medicines, but from them she derived no benefit. The cold and warm bath have been repeatedly employed, but both have been equally unsuccesful.

The long continuance of this complaint with the variety of of symptoms connected with it, rendered it a case of considerable difficulty. I, however, had previously seen such a variety of diseases of these parts, the causes of which could not be very satisfactorily accounted for, but which evidently existed in consequence of a diseased, for the most part diminished, action of the vessels connected with them, that I did not hesitate about its nature; and her pulse being extremely feeble, I thought it proper to proceed on the supposition of this being the principal cause of her complaints.

On the 8th of November, 1807, I prescribed tinct. canth. ℥ß. aq. font ʒvi.; a table-spoonful to be taken four times a day. About a week after she felt slight pain in voiding urine, and I requested her to regulate the doses so as to produce this effect in a slight degree, but always to avoid taking it when the pain became severe.

On the 1st of December, she had a return of her original complaint, for the first time since she began to take the cantharides; and although she thought it was equally severe, yet it did not affect her exactly in the way that it formerly did. The pain formerly seized her suddenly, and to a great extent, and its departure was equally sudden. Now, the grating and darting pain approached gradually, and went off by degrees. No other change had taken place in her complaints. I therefore ordered the cantharides to be continued.

On the 6th, she had a slight return of her complaint, and she suffered some pain from the cantharides in passing water. Eager to relieve herself from the disease, she very inconsiderately took larger doses of the cantharides, and in the evening she was seized with most excruciating pain in voiding urine, accompanied by the most violent hysteria. As she resided a few miles in the country, it was several hours before I saw her, when I immediately ordered the application of cloths dipt in warm water to be made to the abdomen, and at the same time, prescribed a smart cathartic. Before the following morning she was relieved from the pain, but suffered a great deal from general debility.

On the 9th, she recommenced the use of the cantharides in small doses, and informed me that the leucorrhœa had completely disappeared.

On the 11th, she had a return of her old complaint, but thought the pain considerably different in its nature from what it had been.

On the 16th, she had several times since the last report suffered suffered considerably, in consequence of having used the cantharides rather freely; but without any application for the purpose, it gradually left her in a few hours. She uniformly observed, that when the pain or difficulty in voiding urine was severe, she had a slight return of the leucorrhœa, which left her when the pain went off. Her original complaint, compared to what it was in severity, was now very trifling. The cantharides were still continued.

On the 20th, she had suffered considerably from pain in her head, and a sort of feeling as if her face were swelled, particularly after taking the cantharides. As in similar instances of pain in the head, evidently arising from the stomach, I had experienced the happiest effects from moderate doses of an infusion of chamomile flowers, I prescribed this medicine to my patient, and at the same time, desired her to continue the cantharides.

On the 23d, she had experienced great relief from the chamomile, and she is scarcely conscious of the existence of her original complaint. I therefore ordered the cantharides and chamomile to be continued.

On the 1st of January, 1808, this complaint had completely disappeared, and her menstruation, which took place a day or two before, only troubled her about an hour, and the quantity was also very trifling; even less than she ever observed it on former occasions. I ordered the cantharides to be continued.

On the 25th, she had continued to take the cantharides since last report, except twice, when she omitted it for two or three days, and then she had a slight return of her original complaint; and when she took it to produce a good deal of pain, she always had a limpid discharge per vaginam. Her menstruation now again commenced without any pain; and I ordered the cantharides still to be continued.

On the 27th, Menstruation had stopt. It, at this time, had been in greater quantity, and continued much longer than usual. During the last day of it, however, she suffered a good deal of pain from the medicine. I therefore ordered the canth. in small doses.

On the 10th of February, she had no complaint except during sudden changes of the weather, when she had a slight degree of her original disease. I therefore desired the cantharides to be continued.

On the 12th of March, her complaints had entirely disappeared, and in her general health she was much better than ever she recollected having been. She had taken no cantharides for several weeks, and I had not desired her to recommence it; her appearance was now much improved. In September this patient continued perfectly well.

### CASE VII.

Miss ———, aged 28, was in early life, uncommonly stout, healthy, and possessed of an extraordinary share o animation. From sudden exposure to cold, about six years ago, after having heated herself by dancing, she was seized with *rigours*. Medical assistance was sent for, and she apparently recovered; but when her next menstrual period arrived, instead of the flow, as usual, commencing without any troublesome symptom, she was, two days previous to its expected appearance, seized with very excruciating pains in her back, darting down her thighs; and there was a semi-transparent fluid discharged *per vaginam* in considerable quantity. Two days afterward, the acute pain still continuing, menstruation commenced, and in a few hours the pains in her back, &c. abated. Menstruation ceased as usual in four days, and she, in every respect, recovered her health, but pains of increasing severity continued to harass her for ten successive months, at each return of the menstrual period, and then for the first time, she was affected with a regular leucorrhœal discharge. She at that time resided in London, and although most extraordinary quantities of different medicines were daily given her, she derived no benefit from them. About this time her general health began to suffer considerably; and from the repeated failure of these assurances of complete recovery, which she was daily taught to expect, she at length formed a resolution to take no more medicine. She became discontented, of an irritable temper, and was seldom entirely free from violent hysteric affections. She then came to Edinburgh, where some of her relations resided, and was again prevailed on to recommence the use of medicines. Among other prescriptions, she was purged without mercy, but still there was no abatement of her complaints; and at each return of her menstruation, the periods of which still continued regular, the bearing down pains as they are called were so distressing that they almost threw her into a state of delirium. At such times, she made use of immense quantities of laudanum, from which she experienced only momentary relief. She was now obliged to confine herself to bed, a week before, and nearly nearly a fortnight after each menstrual evacuation; during which times her sufferings were indescribable.

When I was desired to see her, I found her in the greatest possible distress, and was informed by her mother of the above circumstances respecting her complaints. She was pale, but not in the least degree emaciated. It was a sort of paleness more frightful than I ever recollect to have seen in any person. Her feet and ankles were almost constantly œdematous, and several of the nails of her toes had separated from her feet; and her hair had almost all fallen off. Her pulse was feeble and irregular. I proposed to try the effects of the tinct. cantharides, conceiving it in this case, to be the only medicine that could be of service to her; but I had considerable difficulty in persuading either the lady or her relations to try any more medicines, as from the disappointment they had already experienced, they expected no benefit from them. She however at last consented, and I prescribed a mixture, each dose of which contained ʒß. of the tinct. cantharid. which I desired her to take thrice a day. She gradually increased the dose, till she could take about ℥ß. of the tincture daily, without suffering the slightest uneasiness in voiding urine; and this quantity she continued to take for the space of five weeks. In the interim her menstrual period arrived, but there was, at this time, no alleviation of her former symptoms. She, on the contrary thought, if possible, that they were aggravated. In a few days after, the effect of the medicine on her urinary organs was felt; but I did not see her till the following day. From the time that this effect was produced till I saw her, she had not diminished the doses of the medicine, although I had desired her to do so when the above symptom became troublesome; and in consequence of this omission, she was now suffering considerable pain. I requested her immediately to desist, prescribed a smart cathartic, and recommended the application of cloths dipt in warm water to the abdomen. Next day, this disagreeable sensation had considerably abated, and was completely removed by the following morning. The leucorrhœal discharge had increased in quantity, which alarmed her very much; and owing to this circumstance, I had again some difficulty in prevailing on her to recommence the medicine. Next return of her menstrual discharge was attended with pains equally severe as formerly; but the following curious occurrence took place at this time, which I had never witnessed on any former occasion, or in any other patient.

Instead of the leucorrhœal discharge being increased in quantity, immediately before and after the flow of the menses, it entirely disappeared one day before, and returned two days after the menses had stopt with increased violence. The doses of the medicine were now taken in very small quantity, as the urinary organs were easily affected by it; and her pulse had now become full and regular. Next menstrual period was preceded by no pain, but it was considerable during the flow, and the quantity of the menstrual fluid was, at this time, truly alarming. This however went off in five days, when the leucorrhœal discharge seemed greatly diminished. The severity of her complaints having now relaxed, I desired her to go into the cold bath thrice a week. She soon after went into the country, and from her habit in using the medicine, I requested of her to use it while she was there. I heard from her about five weeks afterward, when she informed me that her complaints had almost all disappeared, and that her menstruation was not now attended by pain. She still however complained of great general weakness, and said that her appetite was very bad. I desired her to leave off the use of the cantharides, and to substitute for it bark and wine in pretty liberal quantities, and when her appetite was somewhat restored, to add to that rich soup and nourishing diet.

This lady had not, at the end of six months, improved much in strength, nor had her colour in the smallest degree altered for a more healthy appearance, yet her leucorrhœa was gone, and her menstruation had become regular and without pain; and although the hysteric affections were still very troublesome, she could now completely subdue them by the use of pills composed of assafœtida and opium.

This patient had, in September, 1808, completely recovered; her appearance was greatly improved; she could walk a few miles without being at all fatigued, and the hysteric affection only troubled her when her mind was violently agitated.

The cases which I have inserted in this and the previous numbers of your Journal, are only a few of those which I have seen of these complaints, and inserted merely as illustrating the sort of diseases, in addition to those mentioned in my practical treatise on the internal use of cantharides, which may be removed by the well regulated administration of that useful medicine.

*No. 4, St. James's Street.*

CASE

